# Rate-Splitting-Based RF-UWOC Relaying Systems with Hardware Impairments and Interference

**DOI:** 10.3390/e28040458

**Published:** 2026-04-16

**Authors:** Xin Huang, Yeqing Su, Yuehao Qiu, Xusheng Tang, Sai Li

**Affiliations:** 1 GAC Platform Technology Research Institute, Guangzhou Automobile Group Co., Ltd., Guangzhou 511434, China; 2 School of Cyber Science and Engineering, Southeast University, Nanjing 211102, China; 3 School of Communications and Information Engineering, Nanjing University of Posts and Telecommunications, Nanjing 210003, China

**Keywords:** rate-splitting, RF-UWOC, hardware impairments, interference, outage probability

## Abstract

To meet the future demands of high-rate transmission and full-coverage networks, radio frequency–underwater wireless optical communication (RF-UWOC) relaying systems are considered a promising heterogeneous communication architecture. The rate-splitting (RS) scheme, through its power allocation (PA) mechanism, provides a generalized framework for the performance evaluation of such systems. Based on this, this paper analyzes the performance of an RS-based RF-UWOC system under hardware impairments (HIs) and interference. Analytical expressions of the outage probability (OP) and ergodic capacity (EC) for the considered system are formulated within a generalized framework, which encompasses the conventional RF-UWOC system as a special case. The results indicate that the OP and EC are affected by HIs, interference transmit power, the PA coefficients, channel fading, pointing errors (PEs), and detection types of the UWOC link. Furthermore, the asymptotic results for the OP and the diversity gain (DG) are explicitly characterized. For a fixed interference transmit power, the DG is mainly dominated by the channel fading severity, PEs effect, and the detection scheme. When the interference transmit power is comparable to the desired signal power, the system operates in an interference-limited regime, and the DG decreases to zero. It is also revealed that HIs and PA coefficients affect the coding gain but not the DG. Moreover, the existence of an optimal PA scheme improves the reliability of the RS-based system.

## 1. Introduction

Space–air–ground–sea-integrated networks (SAGSINs) are widely recognized as a key architecture for next-generation communication systems [1,2]. In this context, underwater operations, marine environmental monitoring, and maritime Internet-of-Things applications impose ever-increasing demands for high-reliability and high-rate communications. With its capability to support high-throughput underwater links, underwater wireless optical communication (UWOC) serves as an essential solution for enhancing underwater communication performance and facilitates the development of mixed radio frequency (RF)–UWOC communication systems [3]. The RF-UWOC system combines the advantages of mature terrestrial infrastructure in RF communication with the large-capacity characteristic of UWOC links. Compared with conventional UWOC systems, which are limited in transmission distance, the RF-UWOC relaying system significantly extends the effective coverage by introducing RF links for access or backhaul, and exhibits high robustness in challenging underwater turbulence environments. This architecture provides a solid networking foundation for SAGSINs and offers reliable communication support for underwater operations and marine observation [3,4]. In previous studies, in-depth discussions were conducted regarding RF-UWOC system performance, and the motivation and significance of this mixed communication system were clearly explained [3,4,5,6,7,8,9,10,11,12,13,14].

### 1.1. Related Works

With the aid of relaying mechanisms, RF-UWOC systems can substantially extend the transmission distance and support high-rate underwater data transfer via UWOC links. In particular, the studies in [3,4,5,6,7,8] focused on mixed RF-UWOC links applying decode-and-forward (DF) and amplify-and-forward (AF) relaying strategies, where the outage probability (OP), average bit error rate (BER), and ergodic capacity (EC) were reported for reliability evaluation. The secrecy behavior of RF-UWOC relaying systems was further addressed in [9,10], where the secrecy outage probability (SOP) under different fading conditions was derived and analyzed. Moreover, several studies combined satellite links with UWOC links to construct satellite–UWOC relaying systems, in which OP and SOP were comprehensively examined [11,12]. In addition, an RF-UWOC system assisted by an unmanned aerial vehicle (UAV) and based on non-orthogonal multiple access was investigated in [13]. In [14], a reconfigurable intelligent surface (RIS) was incorporated into the RF-UWOC system to enhance its security performance.

Despite the numerous advanced studies on RF-UWOC systems, non-ideal transceiver components in practical deployment scenarios inevitably introduce hardware impairments (HIs), thereby degrading system performance [15]. Moreover, multiple sea-surface nodes share spectrum resources and cause co-channel interference (CCI), which further degrades system performance [16]. In [17,18], the authors analyzed the impact of HIs and interference in other configurations, such as UAV-aided uplink systems. Moreover, in our conference version, an RF-UWOC system impaired by the CCI was investigated [19], where two interference conditions were discussed. However, for RF-UWOC systems, most existing studies assume ideal hardware conditions and ignore the impact of interference, and the performance analysis under the coexistence of both conditions has not been fully studied.

Recently, a rate-splitting (RS) scheme has attracted considerable attention, as it can effectively alleviate the performance degradation caused by HIs or interference and thus enhance the transmission reliability and robustness of the system [20,21]. RS is an advanced physical layer technique that improves wireless network capacity through flexible interference management mechanisms and is considered one of the most promising multiple-access methods in future communication systems. In the RS framework, user information is divided into common and private components and transmitted in a superimposed manner. Upon reception, the common message is decoded first, and its corresponding interference is removed using successive interference cancellation (SIC), followed by the decoding of the private message [22]. By virtue of this decoding mechanism, the RS exhibits strong robustness and adaptability to complex channel environments. Consequently, the RS scheme offers greater design flexibility, such as an adjustable power allocation (PA) mechanism. This advantage is particularly significant in scenarios with HIs, enhanced security requirements, or specific design constraints. At present, the RS has been applied to terahertz (THz) systems, where it effectively improves the outage performance of THz links [23]. Very recently, RS has been introduced into a mixed RF-free space optical (FSO) system [24]. Unlike FSO channels, UWOC links exhibit more challenging physical characteristics inherent to underwater environments. Despite these differences, most prior RS research focuses on multi-user or terrestrial FSO-based relaying scenarios, while the application of RS to RF-UWOC architectures remains unexplored. For such systems, RS introduces a generalized transmission framework, which allows conventional transmission to appear as a special case when the PA coefficient of the common stream equals zero.

### 1.2. Motivations and Contributions

Inspired by previous discussions, this paper investigates the performance of an RS-based RF-UWOC system under the joint effects of HIs and interference, where the RS scheme is adopted to enhance design flexibility and establish a unified framework for performance analysis. To the authors’ knowledge, the joint impact of HIs and interference on RF-UWOC systems, as well as the application of RS in this context, has not yet been investigated. The key contributions of this work are summarized below.

- An RS-based RF-UWOC DF relaying system with HIs and interference is presented, where the RF link follows Nakagami-*m* fading, the interference links are modeled as Rayleigh fading, and the UWOC link experiences exponential-generalized gamma (EGG) fading with pointing errors (PEs).
- The OP and EC of the proposed system are analytically derived within a generalized framework. This framework also covers special cases, including the conventional RF-UWOC without RS and the RF-UWOC system without HIs.
- To gain deeper insights, we derive the asymptotic expressions of the OP. Two interference scenarios are examined, and the corresponding diversity gains (DGs) are obtained. In Case I, with fixed interference transmit power, the DG is jointly dominated by the channel fading parameters, the detection scheme adopted in the UOWC link, and the PEs. In Case II, when the interference transmit power is comparable to the desired signal power, the DG is equal to zero and the OP exhibits an error floor.
- In the RS-based RF-UWOC system, the optimal PA coefficient is determined, which minimizes the system OP and improves overall performance.

### 1.3. Organization and Symbols

The remainder of this paper is structured as below. Section 2 presents the RS-based RF-UWOC DF relaying system, provides details of the signal transmission framework and defines the equivalent signal-to-interference-plus-noise-and-distortion ratio (SINDR). Section 3 introduces the channel statistical models of all links. Based on these fading models, the OP and EC of the system are derived, and several special cases are discussed. The asymptotic behavior under different interference conditions is further analyzed. Furthermore, the optimal PA coefficient is investigated. The numerical results are illustrated and analyzed in Section 4. Section 5 provides the conclusions of this paper. The derivations of the theorems are included in Appendix A, Appendix B and Appendix C. Key parameters and symbols are list in Table 1.

## 2. System Model

**Figure 1:**
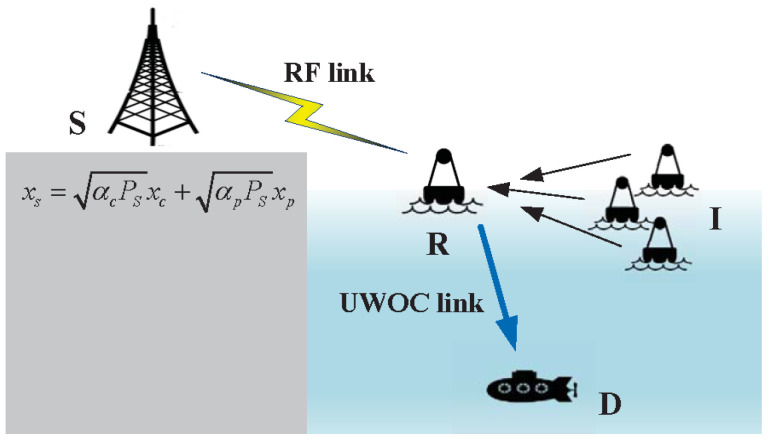
System model.

Figure 1 presents the considered RS-based RF-UWOC relaying scenario under the HIs and CCI. A DF relay (R) positioned at the sea surface assists the information delivery between the source (S) and the undersea destination (D). The first hop (S-R) uses the RF transmission, while the second hop (R-D) adopts UWOC transmission. In this model, S first applies the RS scheme to divide the message into a common stream and a private stream. These streams are transmitted to R through the RF link, where the received signal is affected by CCI from *K* interference nodes (I) with only RF transmission capability. After decoding the received signals, R forwards the common and private streams to D via the UWOC link. Considering the non-ideal characteristics of RF and optical devices in actual systems, all nodes experience HIs. Moreover, each terminal operates with a single antenna, and perfect instantaneous channel state information (CSI) is available for all links.

### 2.1. Signal Transmit

At the first stage, the RS scheme is applied at S. The transmitted message is split into a common component and a private component. The common component is encoded into a common data stream $x_{c}$, and the private component is encoded into a private data stream $x_{p}$, both satisfying $E[|x_{c}{|}^{2}]=E[|x_{p}{|}^{2}]=1$. Accordingly, the transmit signal at S is given by

(1) $$x_{s}=\sqrt{\alpha_{c}P_{S}}x_{c}+\sqrt{\alpha_{p}P_{S}}x_{p}$$

where $P_{S}$ denotes the transmit power at S, and $\alpha_{c}$ and $\alpha_{p}$ represent the PA coefficients for the common and private streams, respectively, satisfying $\alpha_{c}+\alpha_{p}=1$.

Based on this, the received signal at R is formulated as

(2) $$\begin{array}{c} y_{r}=h_{SR}\left(\sqrt{\alpha_{c}P_{S}}x_{c}+\sqrt{\alpha_{p}P_{S}}x_{p}\right)+\sum_{k=1}^{K}\sqrt{P_{I_{k}}}h_{I_{k}R}x_{I_{k}}+\eta_{1}+n_{R} \end{array}$$

where $h_{SR}$ denotes the channel coefficient of the S-R link, $x_{I_{k}}$ stands for the signal satisfying $E[|x_{I_{k}}{|}^{2}]=1$ at the *k*th I, $h_{I_{k}R}$ represents the channel gain from *k*th to R (I-R) link, $P_{I_{k}}$ holds for the transmit power of the *k*th I, $n_{r}∼\mathrm{CN}\left(0,N_{r}\right)$ is the additive white Gaussian Noise (AWGN) at R with zero mean and variance $N_{r}$, $\eta_{1}$ represents the distortion noise resulting from the combined HIs of the S-R and I-R links and is modeled as $\eta_{1}∼\mathrm{CN}\left(0,\Phi_{1}\right)$, with

(3) $$\begin{array}{c} \Phi_{1}=\kappa_{sr}^{2}P_{S}|h_{SR}{|}^{2}+\sum_{k=1}^{K}\kappa_{I_{k}r}^{2}P_{I_{k}}{\left|h_{I_{k}R}\right|}^{2} \end{array}$$

where $\kappa_{sr}$ and $\kappa_{I_{k}r}$ stand for the respective hardware levels of the S-R and I-R links [15]. For simplicity, the HIs from each interference node I to R are assumed to be identical, i.e., $\kappa_{I_{1}r}=\cdots =\kappa_{I_{k}r}=\cdots =\kappa_{Ir}$.

At the second stage, the common and private streams are decoded at R using the DF protocol. Then, R performs electrical-to-optical conversion and transmits the streams to D through the UWOC channel. The received signal at D can be expressed as

(4) $$y_{d}=\eta_{t}^{\frac{r}{2}}h_{RD}^{\frac{r}{2}}\left({\hat{x}}_{s}+\eta_{2}\right)+n_{d}$$

where ${\hat{x}}_{s}$ is the decoded signal, $\eta_{t}$ represents the electro-optical conversion factor, $h_{RD}$ characterizes the channel gain of R-D link, which accounts for underwater optical channel fading and PEs, $\eta_{rd}$ denotes the corresponding distortion noise and is modeled as $\eta_{2}∼\mathrm{CN}\left(0,\kappa_{rd}^{2}P_{R}\right)$ [15], $P_{R}$ stands for the signal power emitted by R, $n_{d}∼\mathrm{CN}\left(0,N_{d}\right)$ is the AWGN at D, and *r* specifies the detection scheme, where r=1 holds for heterodyne detection (HD) and r=2 refers to intensity modulation/direct detection (IM/DD), respectively.

### 2.2. Equivalent SINDR

For the considered DF relaying system, due to the adoption of the RS, both R and D decode the common and private streams separately. As a result, the SINDR expression at each hop contains two parts, namely the common stream part and the private stream part.

Using (2) and the RS strategy, R first decodes the common data stream $x_{c}$ while treating $x_{p}$ as interference. After successful decoding of $x_{c}$, it is removed using the SIC, and $x_{p}$ is subsequently decoded. The SINDRs at R for the common and private streams are obtained, respectively, as

(5) $$\gamma_{sr,c}=\frac{\alpha_{c}\gamma_{1}}{\alpha_{p}\gamma_{1}+\kappa_{sr}^{2}\gamma_{1}+\left(1+\kappa_{Ir}^{2}\right)\gamma_{I}+1}$$

(6) $$\gamma_{sr,p}=\frac{\alpha_{p}\gamma_{1}}{\kappa_{sr}^{2}\gamma_{1}+\left(1+\kappa_{Ir}^{2}\right)\gamma_{I}+1}$$

where $\gamma_{1}=\frac{P_{S}{\left|h_{SR}\right|}^{2}}{N_{r}}={\bar{\gamma }}_{1}{\left|h_{SR}\right|}^{2}$, $\gamma_{I}=\sum_{k=1}^{K}\frac{P_{I_{k}}{\left|h_{I_{k}R}\right|}^{2}}{N_{r}}={\bar{\gamma }}_{I}{\left|h_{IR}\right|}^{2}$ with $|h_{IR}{|}^{2}≜\sum_{k=1}^{K}{\left|h_{I_{k}R}\right|}^{2}$.

Following (4), the SINDRs at D for the common and private streams are expressed, respectively, as

(7) $$\gamma_{rd,c}=\frac{\alpha_{c}\gamma_{2}}{\alpha_{p}\gamma_{2}+\kappa_{rd}^{2}\gamma_{2}+1}$$

(8) $$\gamma_{rd,p}=\frac{\alpha_{p}\gamma_{2}}{\kappa_{rd}^{2}\gamma_{2}+1}$$

where $\gamma_{2}=\frac{\eta_{t}^{r}P_{R}h_{RD}^{r}}{N_{d}}=\varpi_{r}h_{RD}^{r}$, with $\varpi_{r}=\frac{\eta_{t}^{r}P_{R}E{\left[h_{RD}\right]}^{r}}{N_{d}}$ denoting the electrical signal-to-noise ratio (SNR). The parameter $\varpi_{r}$ is obtained from the average SNR ${\bar{\gamma }}_{2}$, which is defined as ${\bar{\gamma }}_{2}=\eta_{t}^{r}P_{R}E\left[h_{rd}^{r}\right]/N_{d}$ [7].

In the present analysis, the DF relaying protocol is adopted, the equivalent SINDRs are characterized as

(9) $$\gamma_{c}=\min\left\{\gamma_{sr,c},\gamma_{rd,c}\right\},\gamma_{p}=\min\left\{\gamma_{sr,p},\gamma_{rd,p}\right\}$$

## 3. Performance Analysis

The statistical properties of all channels are first presented under ideal HIs conditions, which serves as the foundation for deriving the analytical OP and EC for the proposed RS-based RF-UWOC system with HIs and interference. The asymptotic OPs and the corresponding DG under different interference scenarios are examined. The optimal PA coefficient is also obtained in this section.

### 3.1. The Channel Models

Since the SINDR expressions in (5)–(8) depend on $\gamma_{1}$, $\gamma_{I}$, and $\gamma_{2}$, the probability density function (PDF) and cumulative distribution function (CDF) of $\gamma_{Q}$ are first given as follows, where $Q\in \{\gamma_{1},\gamma_{I},\gamma_{2}\}$. Based on these distributions, the CDFs of the SINDRs are then obtained by applying variable transformations and subsequently used to evaluate the system OP and EC.

#### 3.1.1. The RF Channel

For the S-R link, the channel gain follows Nakagami-*m* fading. Accordingly, the PDF and CDF of $\gamma_{1}$ are written as [6]

(10) $$f_{\gamma_{1}}\left(\gamma \right)=\frac{m^{m}}{\Gamma \left(m\right){\bar{\gamma }}_{1}^{m}}\gamma^{m-1}e^{-\frac{m\gamma }{{\bar{\gamma }}_{1}}}$$

(11) $$F_{\gamma_{1}}\left(\gamma \right)=1-\frac{1}{\Gamma (m)}\Gamma \left(m,\frac{m\gamma }{{\bar{\gamma }}_{1}}\right)$$

where *m* specifies the S-R link fading parameter, and Γ(·) and Γ(·,·) refer to the Gamma and incomplete Gamma functions, respectively [25].

#### 3.1.2. The Interference Channels

For each *k*th I-R link, Rayleigh fading is assumed and the channel gains $|h_{I_{k}R}|$ are considered independent and non-identically distributed. Consequently, the PDF and CDF of $\gamma_{I}$ are expressed as [26]

(12) $$f_{\gamma_{I}}\left(\gamma \right)=\sum_{i=1}^{\rho (R)}\sum_{j=1}^{\tau_{i}\left(R\right)}\frac{\chi_{i,j}\left(R\right)\mu_{\langle i\rangle }^{-j}}{{\bar{\gamma }}_{I}^{j}\Gamma \left(j\right)}\gamma^{j-1}e^{-\frac{\gamma }{\mu_{\langle i\rangle }{\bar{\gamma }}_{I}}}$$

(13) $$F_{\gamma_{I}}\left(\gamma \right)=\sum_{i=1}^{\rho (R)}\sum_{j=1}^{\tau_{i}\left(R\right)}\sum_{v=0}^{j-1}\frac{\chi_{i,j}\left(R\right)}{v!{\left(\mu_{\langle i\rangle }{\bar{\gamma }}_{I}\right)}^{v}}\gamma^{v}e^{-\frac{\gamma }{\mu_{\langle i\rangle }{\bar{\gamma }}_{I}}}$$

where $R=\mathrm{diag}(\mu_{1},\mu_{2},\cdots ,\mu_{K})$, ρ(R) stands for the count of distinct diagonal elements of R, $\mu_{\langle 1\rangle }>\mu_{\langle 2\rangle }>\cdots >\mu_{\langle \rho (R)\rangle }$ specify their multiplicities, and $\chi_{i,j}\left(R\right)$ refers to the (i,j)th characteristic coefficient associated with R [26].

#### 3.1.3. The UWOC Channel

For the UWOC channel, EGG fading is assumed. Moreover, we consider the PEs effect. The PDF and CDF expressions of $\gamma_{2}$ are given as [27,28]

(14) $$f_{\gamma_{2}}\left(\gamma \right)=\frac{\omega \xi^{2}}{r\gamma }G_{1,2}^{2,0}\left[\left.\begin{array}{c} {\left(\frac{\gamma }{\lambda^{r}A_{o}^{r}\varpi_{r}}\right)}^{\frac{1}{r}} \end{array}\right|\begin{array}{c} \xi^{2}+1\\ 1,\xi^{2} \end{array}\right]+\frac{\left(1-\omega \right)\xi^{2}}{r\gamma \Gamma (a)}G_{1,2}^{2,0}\left[\left.\begin{array}{c} {\left(\frac{\gamma }{b^{r}A_{o}^{r}\varpi_{r}}\right)}^{\frac{c}{r}} \end{array}\right|\begin{array}{c} \frac{\xi^{2}}{c}+1\\ a,\frac{\xi^{2}}{c} \end{array}\right]$$

(15) $$F_{\gamma_{2}}\left(\gamma \right)=\omega \xi^{2}G_{2,3}^{2,1}\left[\left.\begin{array}{c} {\left(\frac{\gamma }{\lambda^{r}A_{o}^{r}\varpi_{r}}\right)}^{\frac{1}{r}} \end{array}\right|\begin{array}{c} 1,\xi^{2}+1\\ 1,\xi^{2},0 \end{array}\right]+\frac{\left(1-\omega \right)\xi^{2}}{c\Gamma (a)}G_{2,3}^{2,1}\left[\left.\begin{array}{c} {\left(\frac{\gamma }{b^{r}A_{o}^{r}\varpi_{r}}\right)}^{\frac{c}{r}} \end{array}\right|\begin{array}{c} 1,\frac{\xi^{2}}{c}+1\\ a,\frac{\xi^{2}}{c}+1,0 \end{array}\right]$$

where $G_{p,q}^{m,n}\left[\cdot \right]$ represents the Meijer’s G function [25], ω, λ, *a*, *b*, and *c* denote the EGG distributed parameters for characterizing the UWOC channel fading, and the constants $A_{o}$ and ξ model the PEs, as described in [29].

### 3.2. Outage Probability

#### 3.2.1. Exact Results

For the considered RS-based DF relaying system, an outage occurs if either the common or private stream fails to be decoded at R or D. Consequently, the OP corresponds to the probability that the equivalent SINDR of the common stream $\gamma_{c}$ lies below its threshold $\gamma_{th,c}$, or the equivalent SINDR of the private stream $\gamma_{p}$ falls below its threshold $\gamma_{th,p}$, expressed mathematically as

(16) $$\begin{array}{cc} P_{out}= & \mathrm{Pr}(\gamma_{c}<\gamma_{th,c}\;\mathrm{or}\;\gamma_{p}<\gamma_{th,p})\\ = & 1-\mathrm{Pr}(\gamma_{c}>\gamma_{th,c},\gamma_{p}>\gamma_{th,p}) \end{array}$$

Thus, the system requires both components to be successfully decoded, and the system OP is defined as [24]

(17) $$P_{out}=\max\left\{F_{\gamma_{c}}\left(\gamma_{th,c}\right),F_{\gamma_{p}}\left(\gamma_{th,p}\right)\right\}$$

where $F_{\gamma_{c}}\left(\gamma_{th,c}\right)$ and $F_{\gamma_{p}}\left(\gamma_{th,p}\right)$ denote the CDF-based OPs of the common and private components, respectively. To obtain $P_{out}$, the expressions of $F_{\gamma_{c}}\left(\gamma_{th,c}\right)$ and $F_{\gamma_{p}}\left(\gamma_{th,p}\right)$ are calculated separately as follows.

**Theorem** **1.** *By using* (9), *and based on* (5) *and* (7) *as well as the corresponding statistical properties in* (11), (12), *and* (15), *the expression of $F_{\gamma_{c}}\left(\gamma \right)$ is derived as*

(18) $$\begin{array}{c} F_{\gamma_{c}}\left(\gamma \right)=\left\{\begin{array}{c} F_{\gamma_{sr,c}}\left(\gamma \right)+F_{\gamma_{rd,c}}\left(\gamma \right)-F_{\gamma_{sr,c}}\left(\gamma \right)F_{\gamma_{rd,c}}\left(\gamma \right),\;\;\;\;\gamma <\min\left\{\frac{\alpha_{c}}{\alpha_{p}+\kappa_{sr}^{2}},\frac{\alpha_{c}}{\alpha_{p}+\kappa_{rd}^{2}}\right\}\\ 1,\;\;\;otherwise \end{array}\right. \end{array}$$

*where $F_{\gamma_{sr,c}}\left(\gamma \right)$ and $F_{\gamma_{rd,c}}\left(\gamma \right)$ are obtained as*

(19) $$\begin{array}{cc} F_{\gamma_{sr,c}}\left(\gamma \right) & =1-\sum_{i=1}^{\rho (R)}\sum_{j=1}^{\tau_{i}\left(R\right)}\frac{\chi_{i,j}\left(R\right)\vartheta_{1}^{-j}}{\Gamma (j)\Gamma (m)}G_{1,0;1,1;1,1}^{1,0;1,1;0,1}\left[\begin{array}{c} \begin{array}{c} -j\;\\ - \end{array}|\begin{array}{c} \;\;1\\ \;\;j \end{array}|\begin{array}{c} \;\;1-m\;\;\\ 0 \end{array}|\vartheta_{1},\vartheta_{2} \end{array}\right] \end{array}$$

*and*

(20) $$F_{\gamma_{rd,c}}\left(\gamma \right)=\omega \xi^{2}G_{2,3}^{2,1}\left[\left.\begin{array}{c} {\left(\frac{\Xi_{c2}}{\lambda^{r}A_{o}^{r}\varpi_{r}}\right)}^{\frac{1}{r}} \end{array}\right|\begin{array}{c} 1,\xi^{2}+1\\ 1,\xi^{2},0 \end{array}\right]+\frac{\left(1-\omega \right)\xi^{2}}{c\Gamma (a)}G_{2,3}^{2,1}\left[\left.\begin{array}{c} {\left(\frac{\Xi_{c2}}{b^{r}A_{o}^{r}\varpi_{r}}\right)}^{\frac{c}{r}} \end{array}\right|\begin{array}{c} 1,\frac{\xi^{2}}{c}+1\\ a,\frac{\xi^{2}}{c}+1,0 \end{array}\right]$$

*where $\vartheta_{1}=\mu_{\langle i\rangle }{\bar{\gamma }}_{I}\left(1+\kappa_{Ir}^{2}\right)$, $\vartheta_{2}=\frac{{\bar{\gamma }}_{1}}{m\Xi_{c1}}$ with $\Xi_{c1}=\frac{\gamma }{\alpha_{c}-\left(\alpha_{p}+\kappa_{sr}^{2}\right)\gamma }$, $\Xi_{c2}=\frac{\gamma }{\alpha_{c}-\left(\alpha_{p}+\kappa_{rd}^{2}\right)\gamma }$, and $G_{p1,q1:p2,q2:p3,q3}^{m1,0:n2,m2:n3,m3}\left[\cdot ,\cdot \right]$ denotes the bivariate Meijer’s G function (BMGF) [30].*

**Proof.**  See Appendix A. □

By replacing γ with $\gamma_{th,c}$ in (18), the OP of the common component is obtained. The following observations can be made. When $\gamma_{th,c}>\frac{\alpha_{c}}{\alpha_{p}+\kappa_{sr}^{2}}$ or $\gamma_{th,c}>\frac{\alpha_{c}}{\alpha_{p}+\kappa_{rd}^{2}}$, the common stream is always in outage, and the OP equals 1. The CDF-based expressions in (18) are valid when the condition of $\gamma_{th,c}<\min\left\{\frac{\alpha_{c}}{\alpha_{p}+\kappa_{sr}^{2}},\frac{\alpha_{c}}{\alpha_{p}+\kappa_{rd}^{2}}\right\}$ is satisfied.

Likewise, following the derivation in Appendix A, the expression of $F_{\gamma_{p}}\left(\gamma \right)$ can be obtained as

(21) $$\begin{array}{c} F_{\gamma_{p}}\left(\gamma \right)=\left\{\begin{array}{c} F_{\gamma_{sr,p}}\left(\gamma \right)+F_{\gamma_{rd,p}}\left(\gamma \right)-F_{\gamma_{sr,p}}\left(\gamma \right)F_{\gamma_{rd,p}}\left(\gamma \right),\;\;\;\;\gamma <\min\left\{\frac{\alpha_{p}}{\kappa_{sr}^{2}},\frac{\alpha_{p}}{\kappa_{rd}^{2}}\right\}\\ 1,\;\;\;otherwise \end{array}\right. \end{array}$$

where

(22) $$\begin{array}{cc} F_{\gamma_{sr,p}}\left(\gamma \right) & =1-\sum_{i=1}^{\rho (R)}\sum_{j=1}^{\tau_{i}\left(R\right)}\frac{\chi_{i,j}\left(R\right)\vartheta_{1}^{-j}}{\Gamma (j)\Gamma (m)}G_{1,0;1,1;1,1}^{1,0;1,1;0,1}\left[\begin{array}{c} \begin{array}{c} -j\;\\ - \end{array}|\begin{array}{c} \;\;1\\ \;\;j \end{array}|\begin{array}{c} \;\;1-m\;\;\\ 0 \end{array}|\vartheta_{1},\vartheta_{3} \end{array}\right] \end{array}$$

and

(23) $$\begin{array}{cc} F_{\gamma_{rd,p}} & \left(\gamma \right)=\omega \xi^{2}G_{2,3}^{2,1}\left[\left.\begin{array}{c} {\left(\frac{\Xi_{p2}}{\lambda^{r}A_{o}^{r}\varpi_{r}}\right)}^{\frac{1}{r}} \end{array}\right|\begin{array}{c} 1,\xi^{2}+1\\ 1,\xi^{2},0 \end{array}\right]+\frac{\left(1-\omega \right)\xi^{2}}{c\Gamma (a)}G_{2,3}^{2,1}\left[\left.\begin{array}{c} {\left(\frac{\Xi_{p2}}{b^{r}A_{o}^{r}\varpi_{r}}\right)}^{\frac{c}{r}} \end{array}\right|\begin{array}{c} 1,\frac{\xi^{2}}{c}+1\\ a,\frac{\xi^{2}}{c}+1,0 \end{array}\right] \end{array}$$

where $\vartheta_{3}=\frac{{\bar{\gamma }}_{1}}{m\Xi_{p1}}$ with $\Xi_{p1}=\frac{\gamma }{\alpha_{p}-\kappa_{sr}^{2}\gamma }$, and $\Xi_{p2}=\frac{\gamma }{\alpha_{p}-\kappa_{rd}^{2}\gamma }$. Similarly, if $\gamma_{th,p}>\frac{\alpha_{p}}{\kappa_{sr}^{2}}$ or $\gamma_{th,p}>\frac{\alpha_{p}}{\kappa_{rd}^{2}}$, the private stream remains in outage and the OP equals 1. The expressions in (22) and (23) hold only when $\gamma_{th,p}<\min\left\{\frac{\alpha_{p}}{\kappa_{sr}^{2}},\frac{\alpha_{p}}{\kappa_{rd}^{2}}\right\}$.

By combining (17), (18), and (21), the OP of the considered RS-based system is obtained. From this result, it can be observed that the OP is influenced by $\alpha_{c}$. When $\alpha_{c}$ is too small, the common stream may fail to be decoded due to insufficient allocated power. Conversely, when $\alpha_{c}$ is excessively large, the power assigned to the private stream is significantly reduced, which may also lead to decoding failure. Therefore, only within a suitable range of $\alpha_{c}$ can both the common and private streams be reliably decoded. The specific impact of the PA coefficient on system performance will be further discussed in Section 3.4.

Special case: For the system without the RS scheme and considering ideal HIs, i.e., $\alpha_{c}=0$ and $\kappa_{sr}=\kappa_{rd}=\kappa_{Ir}=0$, the expression of $P_{out}$ reduces to

(24) $$\begin{array}{cc} P_{out}= & \sum_{i=1}^{\rho (R)}\sum_{j=1}^{\tau_{i}\left(R\right)}\frac{\chi_{i,j}\left(R\right){\left(\mu_{\langle i\rangle }{\bar{\gamma }}_{I}\right)}^{-j}}{\Gamma (j)\Gamma (m)}G_{1,0;1,1;1,1}^{1,0;1,1;0,1}\left[\begin{array}{c} \begin{array}{c} -j\;\\ - \end{array}|\begin{array}{c} \;\;1\\ \;\;j \end{array}|\begin{array}{c} \;\;1-m\;\;\\ 0 \end{array}|\mu_{\langle i\rangle }{\bar{\gamma }}_{I},\frac{{\bar{\gamma }}_{1}}{m\gamma_{th,p}} \end{array}\right]\\ \; & \times \left(1-\omega \xi^{2}G_{2,3}^{2,1}\left[\left.\begin{array}{c} {\left(\frac{\gamma_{th,p}}{\lambda^{r}A_{o}^{r}\varpi_{r}}\right)}^{\frac{1}{r}} \end{array}\right|\begin{array}{c} 1,\xi^{2}+1\\ 1,\xi^{2},0 \end{array}\right]-\frac{\left(1-\omega \right)\xi^{2}}{c\Gamma (a)}G_{2,3}^{2,1}\left[\left.\begin{array}{c} {\left(\frac{\gamma_{th,p}}{b^{r}A_{o}^{r}\varpi_{r}}\right)}^{\frac{c}{r}} \end{array}\right|\begin{array}{c} 1,\frac{\xi^{2}}{c}+1\\ a,\frac{\xi^{2}}{c}+1,0 \end{array}\right]\right) \end{array}$$

which is consistent with the result in [19].

#### 3.2.2. Asymptotic Results

To better understand the diversity behavior of the system, asymptotic analysis is provided. With the limit of high SNR, the asymptotic OP is approximated as [24]

(25) $$P_{out}^{\infty }=\max\left\{F_{\gamma_{c}}^{\infty }\left(\gamma_{th,c}\right),F_{\gamma_{p}}^{\infty }\left(\gamma_{th,p}\right)\right\}$$

where $F_{\gamma_{c}}^{\infty }\left(\gamma_{th,c}\right)$ and $F_{\gamma_{p}}^{\infty }\left(\gamma_{th,p}\right)$ denote the CDF-based asymptotic OPs of common and private components, respectively. In what follows, we obtain their asymptotic results.

For the common component, by employing (18), $F_{\gamma_{c}}^{\infty }\left(\gamma \right)$ is expressed as [7]

(26) $$F_{\gamma_{c}}^{\infty }\left(\gamma \right)=F_{\gamma_{sr,c}}^{\infty }\left(\gamma \right)+F_{\gamma_{rd,c}}^{\infty }\left(\gamma \right)$$

where $F_{\gamma_{sr,c}}^{\infty }\left(\gamma \right)$ and $F_{\gamma_{rd,c}}^{\infty }\left(\gamma \right)$ denote the asymptotic CDFs of the S-R and R-D links for the common component, respectively.

**Theorem** **2.** *For the S-R link, by letting ${\bar{\gamma }}_{1}\to \infty$, the asymptotic CDF under different interference conditions is given as follows:*

- *Case I: If the interference transmit power ${\bar{\gamma }}_{I}$ is comparable to the desired signal power ${\bar{\gamma }}_{1}$, we have*
(27) $$F_{\gamma_{sr,c}}^{\infty }\left(\gamma \right)=1-\sum_{i=1}^{\rho (R)}\sum_{j=1}^{\tau_{i}\left(R\right)}\frac{\chi_{i,j}\left(R\right)}{\Gamma (j)\Gamma (m)}G_{2,2}^{2,1}\left[\left.\begin{array}{c} \mu_{\langle i\rangle }\left(1+\kappa_{Ir}^{2}\right)m\Xi_{c1} \end{array}\right|\begin{array}{c} 1-j,1\\ 0,m \end{array}\right]$$
- *Case II: If ${\bar{\gamma }}_{I}$ is fixed as a constant, we obtain*
(28) $$F_{\gamma_{sr,c}}^{\infty }\left(\gamma \right)=1-\sum_{i=1}^{\rho (R)}\sum_{j=1}^{\tau_{i}\left(R\right)}\frac{\chi_{i,j}\left(R\right)}{\Gamma (j)\Gamma (m)}\left(\Gamma \left(m\right)\Gamma \left(j\right)-\frac{\Gamma (m+j)}{m}{\left(\frac{\vartheta_{1}}{\vartheta_{2}}\right)}^{m}\right)$$

**Proof.**  See Appendix B. □

For the R-D link, by letting ${\bar{\gamma }}_{2}\to \infty$ in (20), $F_{\gamma_{rd,c}}^{\infty }\left(\gamma \right)$ is given by

(29) $$F_{\gamma_{rd,c}}^{\infty }\left(\gamma \right)=A_{1}{\left(\frac{\Xi_{c2}}{\lambda^{r}A_{o}^{r}\varpi_{r}}\right)}^{\frac{1}{r}}+A_{2}{\left(\frac{\Xi_{c2}}{\lambda^{r}A_{o}^{r}\varpi_{r}}\right)}^{\frac{\xi^{2}}{r}}+A_{3}{\left(\frac{\Xi_{c2}}{b^{r}A_{o}^{r}\varpi_{r}}\right)}^{\frac{ac}{r}}+A_{4}{\left(\frac{\Xi_{c2}}{b^{r}A_{o}^{r}\varpi_{r}}\right)}^{\frac{\xi^{2}}{r}}$$

where $A_{1}=\frac{\omega \xi^{2}\Gamma \left(\xi^{2}-1\right)}{\Gamma (\xi^{2})}$, $A_{2}=\omega \Gamma \left(1-\xi^{2}\right)$, $A_{3}=\frac{\left(1-\omega \right)\xi^{2}\Gamma \left(\frac{\xi^{2}}{c}-a\right)}{c\Gamma \left(\frac{\xi^{2}}{c}+1-a\right)\Gamma \left(1+a\right)}$, and $A_{4}=\frac{\left(1-\omega \right)\Gamma \left(\frac{\xi^{2}}{c}\right)\Gamma \left(a-\frac{\xi^{2}}{c}\right)}{\Gamma \left(a\right)\Gamma \left(\frac{\xi^{2}}{c}+1\right)}$.

Combining (27)/(28) and (29) and evaluating at $\gamma =\gamma_{th,c}$ yields the asymptotic OP for the common component. Note that the asymptotic OP takes the form in (26) if $\gamma_{th,c}<\min\left\{\frac{\alpha_{c}}{\alpha_{p}+\kappa_{sr}^{2}},\frac{\alpha_{c}}{\alpha_{p}+\kappa_{rd}^{2}}\right\}$; otherwise, it equals 1.

Similarly, for the private component, $F_{\gamma_{p}}^{\infty }\left(\gamma \right)$ with fixed ${\bar{\gamma }}_{I}$ is formulated as

(30) $$F_{\gamma_{p}}^{\infty }\left(\gamma \right)=F_{\gamma_{sr,p}}^{\infty }\left(\gamma \right)+F_{\gamma_{rd,p}}^{\infty }\left(\gamma \right)$$

where

(31) $$F_{\gamma_{sr,p}}^{\infty }\left(\gamma \right)=1-\sum_{i=1}^{\rho (R)}\sum_{j=1}^{\tau_{i}\left(R\right)}\frac{\chi_{i,j}\left(R\right)}{\Gamma (j)\Gamma (m)}\left(\Gamma \left(m\right)\Gamma \left(j\right)-\frac{\Gamma (m+j)}{m}{\left(\frac{\vartheta_{1}}{\vartheta_{3}}\right)}^{m}\right)$$

and

(32) $$F_{\gamma_{rd,p}}^{\infty }\left(\gamma \right)=A_{1}{\left(\frac{\Xi_{p2}}{\lambda^{r}A_{o}^{r}\varpi_{r}}\right)}^{\frac{1}{r}}+A_{2}{\left(\frac{\Xi_{p2}}{\lambda^{r}A_{o}^{r}\varpi_{r}}\right)}^{\frac{\xi^{2}}{r}}+A_{3}{\left(\frac{\Xi_{p2}}{b^{r}A_{o}^{r}\varpi_{r}}\right)}^{\frac{ac}{r}}+A_{4}{\left(\frac{\Xi_{p2}}{b^{r}A_{o}^{r}\varpi_{r}}\right)}^{\frac{\xi^{2}}{r}}$$

Taking (31) and (32) in (30) and evaluating at $\gamma =\gamma_{th,p}$ yields the asymptotic OP for the private component.

#### 3.2.3. Diversity Gains

Through the above asymptotic analysis, the variation trends of the system performance can be revealed, and the effects of key parameters, such as channel fading, detection schemes, interference, HIs, and PA coefficients, on the system diversity performance can be further analyzed. At high SNR, the asymptotic OP can be expressed as $P_{out}^{\infty }={\left(G_{c}\bar{\gamma }\right)}^{-G_{d}}$[15], where $\bar{\gamma }$ is the average SNR, and $G_{c}$ and $G_{d}$ denote the coding gain (CG) and DG, respectively, characterizing the horizontal shift and slope of the curve.

Consequently, for Case I, when the interference transmit power ${\bar{\gamma }}_{I}$ is fixed, the DG is obtained from the asymptotic results in (26), (28), (29), (30), (31), and (32) as

(33) $$\begin{array}{c} G_{d}=\min\left\{m,\frac{1}{r},\frac{ac}{r},\frac{\xi^{2}}{r}\right\} \end{array}$$

It follows that, in this case, the interference level, HIs level, and PA coefficients affect the CG rather than the DG. For instance, higher values of $\kappa_{sr}$, $\kappa_{Ir}$, and $\kappa_{rd}$ reduce $G_{c}$, which indicates more severe HIs and consequently increases the OP. In contrast, the channel fading parameters (i.e., *m*, ac), the detection scheme employed in the UWOC link (i.e., *r*), and the PEs parameter (i.e., ξ) dominate the DG of the system.

In Case II, if ${\bar{\gamma }}_{I}$ is comparable to the desired signal power, i.e., ${\bar{\gamma }}_{I}={\bar{\gamma }}_{1}$, the DG is obtained from the asymptotic results in (26), (27), (29), (30), (31), and (32) as $G_{d}=\min\left(0,1/r,ac/r,\xi^{2}/r\right)=0$, which implies an interference-limited error floor. In this regime, interference becomes the dominant term affecting system performance, while other parameters, such as the HIs, PA coefficients, PEs, channel fading, and detection parameters, affect only the CG.

### 3.3. Ergodic Capacity

For the RS-based RF-UWOC DF relaying system, two independent streams are decoded sequentially using SIC. Therefore, the overall EC equals the sum of the capacities of the common and private components and is defined as

(34) $$\bar{C}={\bar{C}}_{c}+{\bar{C}}_{p}$$

where

(35) $${\bar{C}}_{\epsilon }=\frac{1}{2}\min\left\{{\bar{C}}_{sr,\epsilon },{\bar{C}}_{rd,\epsilon }\right\}$$

with ϵ∈{c,p}, and ${\bar{C}}_{\hbar ,\epsilon }$ is obtained as ${\bar{C}}_{\hbar ,\epsilon }=\frac{1}{\ln(2)}\int_{0}^{\infty }\frac{1-F_{\hbar ,\epsilon }\left(\gamma \right)}{1+\gamma }d\gamma$ [24], where ℏ∈{sr,rd}. The presence of HIs complicates the derivation of an analytical framework for the EC of the proposed system, as reported in [15,18]. Therefore, a numerical integration approach is employed to approximate the EC. In the following, expressions for ${\bar{C}}_{sr,c}$, ${\bar{C}}_{rd,c}$, ${\bar{C}}_{sr,p}$, and ${\bar{C}}_{rd,p}$ are provided.

**Theorem** **3.** *By employing the numerical integration approach, the expression of ${\bar{C}}_{sr,c}$ can be derived as*

(36) $${\bar{C}}_{sr,c}=\frac{1}{\ln(2)}\sum_{n=0}^{M}w_{n}H_{1}\left(x_{n}\right)$$

*where M denotes the number of Gauss–Laguerre integration points, $w_{n}$ and $x_{n}$ represent the weight factor and the zeros of the Laguerre polynomials, respectively, which are provided in Table 25.9 of [31], and $H_{1}\left(x_{n}\right)$ is given by*

(37) H1(xn)=∑s=0m−1∑t=0s∑i=1ρ(R)∑j=1τi(R)stχi,j(R)ϑ1−jΓ(j)s!Γ(t+j)(1ϑ1+mxnγ¯1)t+j1+(αp+κsr2)xn1+(1+κsr2)xnG0,11,0mxnγ¯1−sαc(1+(αp+κsr2)xn)2

**Proof.**  See Appendix C. □

Likewise, following a similar approach to Appendix C, the expression of ${\bar{C}}_{rd,c}$ is given by

(38) $${\bar{C}}_{rd,c}=\frac{1}{\ln(2)}\sum_{n=0}^{M}w_{n}H_{2}\left(x_{n}\right)$$

where

(39) $$\begin{array}{c} H_{2}\left(x_{n}\right)=\left(1-\omega \xi^{2}G_{2,3}^{2,1}\left[\left.\begin{array}{c} {\left(\frac{x_{n}}{\lambda^{r}A_{o}^{r}\varpi_{r}}\right)}^{\frac{1}{r}} \end{array}\right|\begin{array}{c} 1,\xi^{2}+1\\ 1,\xi^{2},0 \end{array}\right]-\frac{\left(1-\omega \right)\xi^{2}}{c\Gamma (a)}G_{2,3}^{2,1}\left[\left.\begin{array}{c} {\left(\frac{x_{n}}{b^{r}A_{o}^{r}\varpi_{r}}\right)}^{\frac{c}{r}} \end{array}\right|\begin{array}{c} 1,\frac{\xi^{2}}{c}+1\\ a,\frac{\xi^{2}}{c}+1,0 \end{array}\right]\right)\\ \times \frac{1+\left(\alpha_{p}+\kappa_{rd}^{2}\right)x_{n}}{1+\left(1+\kappa_{rd}^{2}\right)x_{n}}\frac{\alpha_{c}}{{\left(1+\left(\alpha_{p}+\kappa_{rd}^{2}\right)x_{n}\right)}^{2}} \end{array}$$

With the help of (36) and (38), the EC for the common component can be obtained.

Similarly, the EC for the private component is computed as

(40) $${\bar{C}}_{p}=\frac{1}{2}\min\left\{{\bar{C}}_{sr,p},{\bar{C}}_{rd,p}\right\}$$

where ${\bar{C}}_{sr,p}=\frac{1}{\ln(2)}\sum_{n=0}^{M}w_{n}H_{3}\left(x_{n}\right)$ and ${\bar{C}}_{rd,p}=\frac{1}{\ln(2)}\sum_{n=0}^{M}w_{n}H_{4}\left(x_{n}\right)$, with $H_{3}\left(x_{n}\right)$ and $H_{4}\left(x_{n}\right)$ derived as

(41) H3(xn)=∑s=0m−1∑t=0s∑i=1ρ(R)∑j=1τi(R)stχi,j(R)ϑ1−jΓ(j)s!Γ(t+j)(1ϑ1+mxnγ¯1)t+j1+κsr2xn1+(αp+κsr2)xnG0,11,0mxnγ¯1−sαp(1+κsr2xn)2

and

(42) $$\begin{array}{c} H_{4}\left(x_{n}\right)=\left(1-\omega \xi^{2}G_{2,3}^{2,1}\left[\left.\begin{array}{c} {\left(\frac{x_{n}}{\lambda^{r}A_{o}^{r}\varpi_{r}}\right)}^{\frac{1}{r}} \end{array}\right|\begin{array}{c} 1,\xi^{2}+1\\ 1,\xi^{2},0 \end{array}\right]-\frac{\left(1-\omega \right)\xi^{2}}{c\Gamma (a)}G_{2,3}^{2,1}\left[\left.\begin{array}{c} {\left(\frac{x_{n}}{b^{r}A_{o}^{r}\varpi_{r}}\right)}^{\frac{c}{r}} \end{array}\right|\begin{array}{c} 1,\frac{\xi^{2}}{c}+1\\ a,\frac{\xi^{2}}{c}+1,0 \end{array}\right]\right)\\ \times \frac{1+\kappa_{rd}^{2}x_{n}}{1+\left(\alpha_{p}+\kappa_{rd}^{2}\right)x_{n}}\frac{\alpha_{p}}{{\left(1+\kappa_{rd}^{2}x_{n}\right)}^{2}} \end{array}$$

### 3.4. Power Allocation Optimization

In RS-based systems, properly adjusting the PA coefficient can effectively improve the system performance. When $\alpha_{c}$ is small, the common stream suffers from insufficient transmit power and cannot be reliably decoded, leading to system outage. As $\alpha_{c}$ increases, the reliability of the common component is enhanced while the private stream still retains sufficient power. In this region, proper PA between the common and private streams reduces the OP, allowing it to reach its minimum value. However, when $\alpha_{c}$ is further increased, the transmit power allocated to the private stream is significantly reduced, which degrades its decoding performance and results in an increased OP. Therefore, there is an optimal value of $\alpha_{c}$ to effectively enhance overall transmission performance. From the asymptotic results in (28), (29), (31), and (32), the OP is related to the parameters $\Xi_{c1}$, $\Xi_{c2}$, $\Xi_{p1}$, and $\Xi_{p2}$. According to (25), the optimization problem is stated as

(43) $$\begin{array}{cc} \min\mathrm{imize} & \max\{\Upsilon_{c},\Upsilon_{p}\}\\ \;\;\;\;\;\;\;\;\;s.t. & \alpha_{c}+\alpha_{p}=1 \end{array}$$

where $\Upsilon_{c}=\min\left\{\Xi_{c1},\Xi_{c2}\right\}$ and $\Upsilon_{p}=\min\left\{\Xi_{p1},\Xi_{p2}\right\}$. Since $\Xi_{c1}$, $\Xi_{c2}$, $\Xi_{p1}$, and $\Xi_{p2}$ are all functions of $\alpha_{c}$, the objective function can be minimized by setting $\Upsilon_{c}=\Upsilon_{p}$ [23]. Based on this observation, the optimal PA coefficient is calculated as

(44) $$\alpha_{c}=\frac{\gamma_{th,c}+\gamma_{th,c}\gamma_{th,p}}{\gamma_{th,c}+\gamma_{th,p}+\gamma_{th,c}\gamma_{th,p}}$$

which can also be expressed as the ratio $\frac{\alpha_{c}}{\alpha_{p}}=\frac{\gamma_{th,c}\left(\gamma_{th,p}+1\right)}{\gamma_{th,p}}$. This result reveals that the optimal PA coefficient is solely a function of the target threshold and does not depend on the HI parameters.

## 4. Numerical Results

This section provides numerical demonstrations using the derived analytical expressions, with results verified through Monte Carlo simulations. The number of simulation runs is set to $10^{5}$. The BMGF can be numerically computed in MATLAB or MATHEMATICA using a contour integral-based numerical approach ([30] Table II). Three representative parameter sets are adopted to characterize the UWOC channel fading under different bubble levels (BLs) and temperature gradient (TG) conditions, as listed in Table 2 (these UWOC channel parameters are obtained by fitting experimental data from laboratory water-tank measurements and are used to characterize the severity of underwater optical turbulence fading (see [27] for more details)). Unless otherwise specified, the parameters are set as follows: $\gamma_{th,c}=\gamma_{th,p}=\gamma_{th}=1$ dB, K=3, $\alpha_{c}=0.7$, $A_{o}=0.044$ (the PE parameters $A_{o}$ and ξ depend on the optical beam width $w_{b}$, the receiver aperture $a_{r}$, and the jitter variance $\sigma_{s}$. In this work, $w_{b}=1$ and $a_{r}=0.15$ are adopted as in [29], yielding $A_{o}=0.044$. The parameter $\sigma_{s}$ characterizes the severity of PEs; a larger $\sigma_{s}$ results in smaller ξ and thus more severe PEs), and the fading parameters of UWOC are ω=0.2130, λ=0.3291, a=1.4299, b=1.1817, and c=17.1984. The hardware levels are assumed to be identical for all links, i.e., $\kappa_{sr}=\kappa_{Ir}=\kappa_{rd}=\kappa$. Moreover, the average SNRs of the S-R and R-D links are assumed to be equal, i.e., ${\bar{\gamma }}_{1}={\bar{\gamma }}_{2}=\bar{\gamma }$.

**Figure 2:**
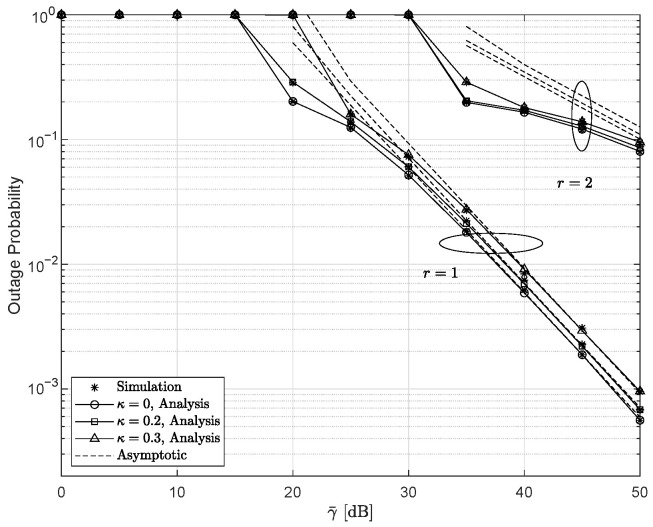
Outage probability versus $\bar{\gamma }$ for various κ and *r* settings, with ξ=10.1188, m=4, and ${\bar{\gamma }}_{I}=0$ dB.

Figure 2 illustrates the OP and its asymptotic behavior as functions of $\bar{\gamma }$ for different values of κ and *r*, with ξ=10.1188, m=4, and ${\bar{\gamma }}_{I}=0$ dB. As expected, it is observed that the IM/DD yields an inferior performance compared with the HD. As the hardware level decreases, the OP degrades accordingly, while ideal hardware consistently outperforms the non-ideal case. Moreover, the results reveal that the detection type governs the slope of the OP curves, namely the DG, whereas the level of HIs primarily affects the CG without altering the DG.

**Figure 3:**
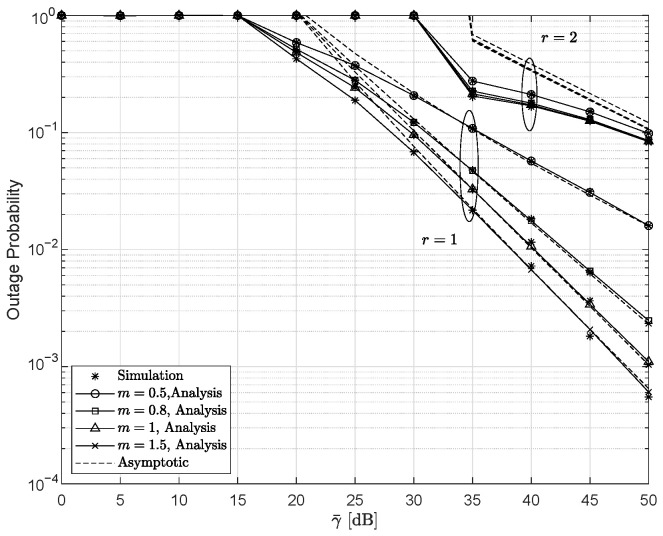
Outage probability versus $\bar{\gamma }$ for several *r* and *m* values, with ξ=5.0594, κ=0.1, r=1, and ${\bar{\gamma }}_{I}=5$ dB.

Considering different channel fading conditions and detection schemes, Figure 3 depicts the OP and its asymptotic results versus $\bar{\gamma }$ with ξ=5.0594, κ=0.1, r=1, and ${\bar{\gamma }}_{I}=5$ dB. The validity of the derived DG in (33) is confirmed by the asymptotic analysis. For the HD scheme (r=1), when the fading parameter *m* takes relatively small values (e.g., m=0.5 or m=0.8), the DG is governed by *m*. As the value of *m* increases to 1 or 1.5, the DG becomes dependent on 1/r=1. In contrast, under the IM/DD scheme, the DG remains unchanged as the value of *m* increases from 0.5 to 1.5. This observation implies that, at high SNRs, system performance is primarily constrained by channel fading characteristics and the detection scheme employed in the UWOC link.

**Figure 4:**
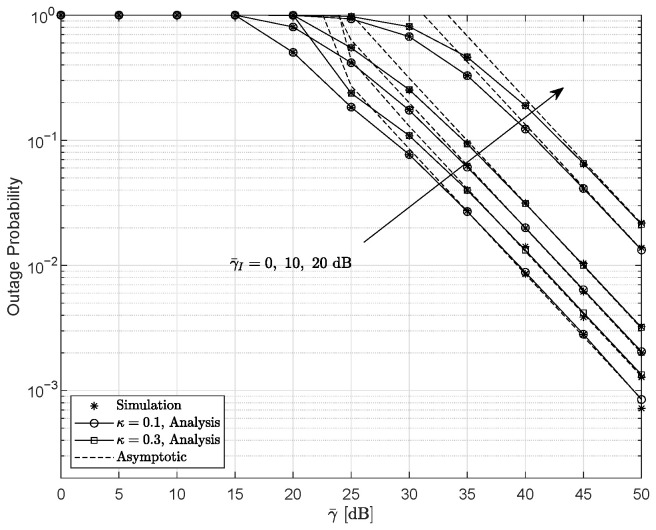
Outage probability as a function of $\bar{\gamma }$ for various ${\bar{\gamma }}_{I}$ and κ configurations for ξ=2.5297, r=1, and m=1.

Figure 4 shows the effects of interference and HIs on the OP with ξ=2.5297, r=1, and m=1. As expected, the outage performance degrades as ${\bar{\gamma }}_{I}$ increases. Moreover, when the hardware level increases from κ=0.1 to κ=0.3, the OP becomes higher, indicating more severe performance loss. For this parameter setting, the DG is given by $\min\{m,\frac{1}{r},\frac{ac}{r},\frac{\xi^{2}}{r}\}=1$ and remains unaffected by either the interference transmit power or the HI level.

**Figure 5:**
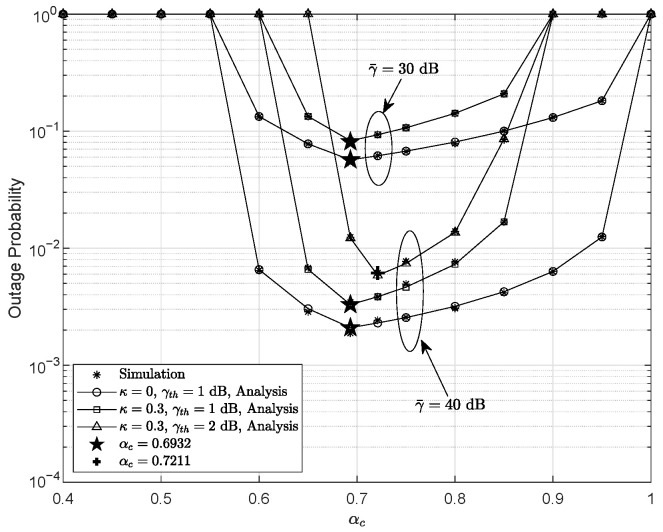
Outage probability as a function of $\alpha_{c}$ for several $\bar{\gamma }$, κ, and $\gamma_{th}$ values, with m=3, r=1, ξ=3.3729, and ${\bar{\gamma }}_{I}=0$ dB.

Figure 5 depicts the OP versus $\alpha_{c}$ for different values of $\bar{\gamma }$, κ, and $\gamma_{th}$, with m=3, r=1, ξ=3.3729, and ${\bar{\gamma }}_{I}=0$ dB. One can observe that the OP decreases as $\bar{\gamma }$ increases. Compared to the ideal hardware case, the presence of HIs leads to noticeable performance degradation. More importantly, the OP first decreases and then increases as $\alpha_{c}$ grows, which reveals a clear U-shaped behavior and indicates the existence of an optimal $\alpha_{c}$. When $\alpha_{c}$ takes a small value, the power assigned to the common component is inadequate. As a result, the maximum achievable SINDR at the receiver does not reach the required threshold, i.e., $\gamma_{th,c}>\min\left(\frac{\alpha_{c}}{\alpha_{p}+\kappa_{sr}^{2}},\frac{\alpha_{c}}{\alpha_{p}+\kappa_{rd}^{2}}\right)=\frac{\alpha_{c}}{\alpha_{p}+\kappa^{2}}$. In this case, the common stream cannot be decoded successfully, leading to system outage. As $\alpha_{c}$ increases, the condition $\gamma_{th,c}<\frac{\alpha_{c}}{\alpha_{p}+\kappa^{2}}$ is satisfied and successful decoding of the common component becomes possible. Meanwhile, the power allocated to the private stream remains sufficient, such that $\gamma_{th,p}<\min\left(\frac{\alpha_{p}}{\kappa_{sr}^{2}},\frac{\alpha_{p}}{\kappa_{rd}^{2}}\right)=\frac{\alpha_{p}}{\kappa^{2}}$. However, when $\alpha_{c}$ exceeds a certain threshold, the value of $\alpha_{p}$ reduces to a level that no longer supports private stream decoding, i.e., $\gamma_{th,p}>\frac{\alpha_{p}}{\kappa^{2}}$, which again results in system outage. Therefore, in the RS-based RF-UWOC system, the OP is affected by the variation in the PA coefficients. As shown in Figure 5, the optimal $\alpha_{c}$ equals 0.6932 for $\gamma_{th}=1$ dB and 0.7211 for $\gamma_{th}=2$ dB, which is consistent with the expression derived in (44). In general, in practical system design, the value of $\alpha_{c}$ is typically set to be slightly larger than $\alpha_{p}$ to first ensure the successful decoding of the common stream, and then guarantee reliable decoding of the private stream.

**Figure 6:**
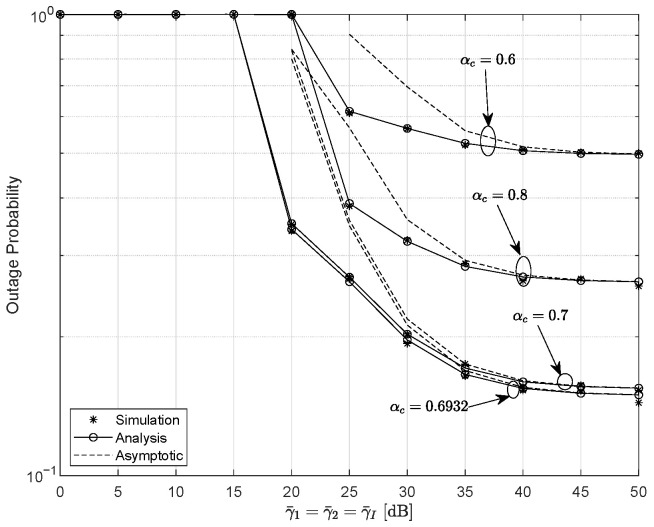
The variation in the outage probability versus ${\bar{\gamma }}_{I}$ for different values of $\alpha_{c}$, with $\gamma_{th}=1$ dB, m=4, and ξ=5.0594.

In the scenario where the interference transmit power ${\bar{\gamma }}_{I}$ approaches the desired signal power ${\bar{\gamma }}_{1}$, Figure 6 show the plot for OP for different values of $\alpha_{c}$. The parameters are set as m=4 and ξ=5.0594. According to the previous analysis, the optimal PA coefficient is $\alpha_{c}=0.6932$ for $\gamma_{th}=1$ dB. As expected, the optimal outage performance occurs at $\alpha_{c}=0.6932$, yielding up to 69% and 47% improvements in system reliability compared with the cases of $\alpha_{c}=0.6$ and $\alpha_{c}=0.8$, respectively. Additionally, when both the interference and signal powers are in the high-SNR regime, the system becomes interference-limited. As a result, the OP exhibits an error floor and the DG reduces to zero. Therefore, interference management or appropriate power control is required in practical deployments.

**Figure 7:**
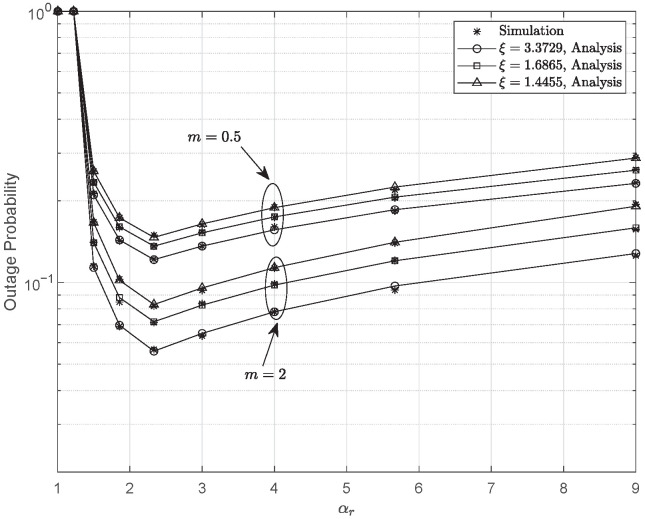
Outage probability versus $\alpha_{r}$ for various values of ξ and *m*, when ${\bar{\gamma }}_{1}={\bar{\gamma }}_{2}=30$ dB, ${\bar{\gamma }}_{I}=0$ dB, r=1, and κ=0.

Defining $\alpha_{c}/\alpha_{p}=\alpha_{r}$, the OP versus $\alpha_{r}$ for various *m* and ξ settings is plotted in Figure 7. The parameters are configured as ${\bar{\gamma }}_{1}={\bar{\gamma }}_{2}=30$ dB, ${\bar{\gamma }}_{I}=0$ dB, r=1, and κ=0. It is observed that the outage performance deteriorates when $\alpha_{r}$ is either too small or too large. This also suggests that the PA coefficients should be carefully selected in practical system design. An appropriate $\alpha_{r}$ can balance the decoding reliability of the common and private streams, thereby reducing the OP. The optimal value of $\alpha_{r}$ is observed to be equal to $1+\gamma_{th}$, as predicted by (44). With larger *m*, the quality of the S-R channel improves, which benefits the overall system performance. Similarly, a higher ξ mitigates the severity of PEs, producing a better outage performance.

**Figure 8:**
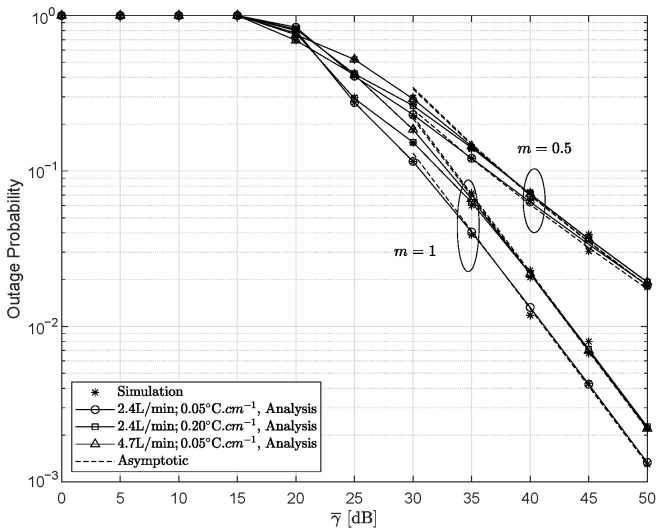
Outage probability as a function of $\bar{\gamma }$ under different fading conditions, when ${\bar{\gamma }}_{I}=5$ dB, κ=0.2, ξ=2.5297, and r=1.

Figure 8 presents the OP and its asymptotic results as functions of $\bar{\gamma }$ for several fading settings of the RF and UWOC, with ${\bar{\gamma }}_{I}=5$ dB, κ=0.2, ξ=2.5297, and r=1. The UWOC fading parameters are set according to Table 2. Under strong fading conditions, the OP increases by approximately 69% at $\bar{\gamma }=50$ dB, which indicates a degradation in system reliability. It is observed that these parameters only affect the CG of the system. In the high-SNR regions, the outage performance remains dominated by the parameter *m*.

**Figure 9:**
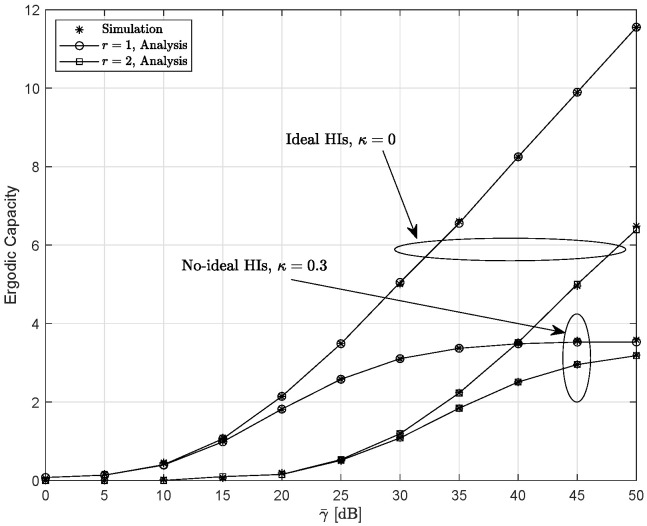
The variation in the ergodic capacity under different HIs conditions for different detection schemes when ${\bar{\gamma }}_{I}=0$ dB, ξ=2.5297, and m=1.

Figure 9 presents the EC for the ideal HIs system and the non-ideal HIs system for ${\bar{\gamma }}_{I}=0$ dB, ξ=2.5297, and m=1. One can notice that the EC of the ideal HIs system increases with the SNR, whereas the EC of the non-ideal HIs system exhibits an error floor at the high SNRs. This behavior arises because HIs limit the achievable performance of the system. In addition, the EC obtained with the HD scheme exceeds that of the IM/DD scheme.

**Figure 10:**
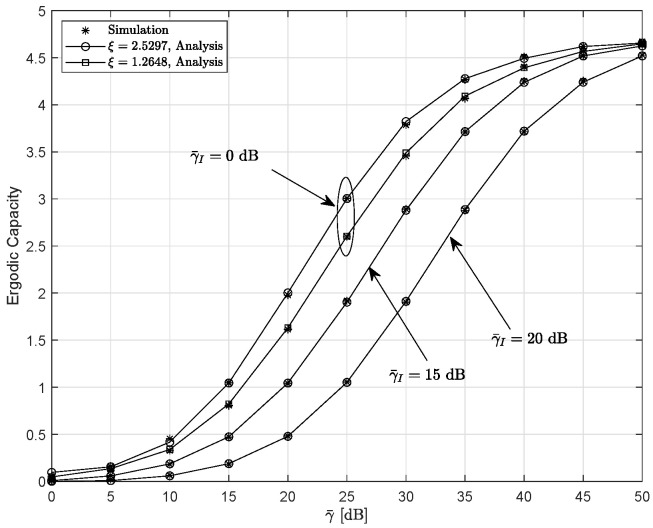
Ergodic capacity versus $\bar{\gamma }$ for the different value of ${\bar{\gamma }}_{I}$ and ξ when κ=0.2, m=2, and r=1.

Figure 10 plots the EC versus $\bar{\gamma }$ for different parameters of ξ and ${\bar{\gamma }}_{I}$. The other parameters are set as κ=0.2, m=2, and r=1. The EC shows an increasing trend with respect to ξ. In contrast, an increase in the interference transmit power degrades the EC.

## 5. Conclusions

This paper investigated an RS-based RF-UWOC DF relaying system in the presence of HIs and CCI. The OP and EC were derived and analyzed under different PA coefficients, interference and hardware levels, fading parameters, detection schemes, and PEs. Moreover, the asymptotic behaviors under various interference scenarios was further characterized. The results demonstrated that when the interference transmit power was comparable to the signal power, the system became interference-limited and the DG reduced to zero. Under fixed interference transmit power, the system DG was primarily governed by the fading parameters, detection scheme, and PEs parameter of the UWOC link, whereas the remaining parameters affected only the CG. For the RS-based system, an optimal PA coefficient was identified that maximizes overall system performance. The analysis was conducted under the assumptions of ideal CSI and perfect SIC. In future work, these practical factors will be taken into account, with a particular emphasis on conducting practical experimental validation, to obtain a more comprehensive analysis. This will provide valuable insights for the implementation of real-world applications, such as underwater sensor networks and ocean monitoring systems.

## Figures and Tables

**Table 1:** Key parameters and symbols.

Key Parameters and Symbols	Significance
$\alpha_{c}$, $\alpha_{p}$	PA coefficients for the common and private streams
$\eta_{1}$, $\eta_{2}$	Distortion noises for the RF link and the UWOC link
*K*	Number of interference
*m*	Channel fading parameter of the RF link
ω, λ, *a*, *b*, *c*	Channel fading parameters of the UWOC link
*r*	Detection schemes
$A_{o}$, ξ	PEs parameters
Γ(·)	Gamma function
Γ(·,·)	Incomplete Gamma function
$G_{p,q}^{m,n}\left[\cdot \right]$	Meijer’s G function
$G_{p1,q1:p2,q2:p3,q3}^{m1,0:n2,m2:n3,m3}\left[\cdot ,\cdot \right]$	Bivariate Meijer’s G function
$f_{\gamma }\left(\cdot \right)$, $F_{\gamma }\left(\cdot \right)$	Probability distribution function and cumulative distribution function of γ

**Table 2:** UWOC channel fading parameters for different BL and TG.

Fading Severity	BL (L/min)	TG (°C·${\mathrm{cm}}^{-1}$)	EGG Distribution Parameters
Weak	2.4	0.05	ω=0.2130, λ=0.3291, a=1.4299, b=1.1817, c=17.1984
Moderate	2.4	0.20	ω=0.1665, λ=0.1207, a=0.1559, b=1.5216, c=22.8754
Strong	4.7	0.05	ω=0.4201, λ=0.4580, a=1.0421, b=1.5768, c=35.9424

## Data Availability

The data are contained within this article.

## Appendix A. Proof of the Expressions for $F_{\gamma_{sr,c}}\left(\gamma \right)$ and $F_{\gamma_{rd,c}}\left(\gamma \right)$

Based on (5), $F_{\gamma_{sr,c}}\left(\gamma \right)$ is written as

(A1) $$\begin{array}{c} F_{\gamma_{sr,c}}\left(\gamma \right)=\mathrm{Pr}\left[\gamma_{1}<\frac{\gamma_{I}\left(1+\kappa_{Ir}^{2}\right)\gamma +\gamma }{\alpha_{c}-\left(\alpha_{p}+\kappa_{sr}^{2}\right)\gamma }\right] \end{array}$$

Accordingly, by conditioning on $\gamma_{I}$, (A1) can be rewritten as

(A2) $$F_{\gamma_{sr,c}}\left(\gamma \right)=\int_{0}^{\infty }F_{\gamma_{1}}\left(\frac{x(1+\kappa_{Ir}^{2})\gamma +\gamma }{\alpha_{c}-\left(\alpha_{p}+\kappa_{sr}^{2}\right)\gamma }\right)f_{\gamma_{I}}\left(x\right)dx$$

First, by substituting (11) and (12) into (A2), and applying ([32] Equations (8.4.3/1) and (8.4.16/2)), the exponential and incomplete gamma functions can be expressed in terms of Meijer’s G functions as

(A3) $$\begin{array}{c} e^{-\frac{x}{\mu_{\langle i\rangle }{\bar{\gamma }}_{I}}}=G_{0,1}^{1,0}\left[\left.\begin{array}{c} \frac{x}{\mu_{\langle i\rangle }{\bar{\gamma }}_{I}} \end{array}\right|\begin{array}{c} -\\ 0 \end{array}\right] \end{array}$$

(A4) $$\begin{array}{c} \Gamma \left(m,\frac{m\gamma (1+\left(1+\kappa_{Ir}^{2}\right)x)}{{\bar{\gamma }}_{1}\left(\alpha_{c}-\left(\alpha_{p}+\kappa_{sr}^{2}\right)\gamma \right)}\right)=G_{1,2}^{2,0}\left[\left.\begin{array}{c} \frac{m\gamma (1+\left(1+\kappa_{Ir}^{2}\right)x)}{{\bar{\gamma }}_{1}\left(\alpha_{c}-\left(\alpha_{p}+\kappa_{sr}^{2}\right)\gamma \right)} \end{array}\right|\begin{array}{c} 1\\ 0,m \end{array}\right] \end{array}$$

Next, expanding these functions according to their definitions ([33] Equation (07.34.02.0001.01)) allows $F_{\gamma_{sr,c}}\left(\gamma \right)$ to be expressed as

(A5) $$\begin{array}{cc} F_{\gamma_{sr,c}}\left(\gamma \right) & =1-\sum_{i=1}^{\rho (R)}\sum_{j=1}^{\tau_{i}\left(R\right)}\frac{\chi_{i,j}\left(R\right){\left(\mu_{\langle i\rangle }{\bar{\gamma }}_{I}\right)}^{-j}}{\Gamma (j)\Gamma (m)}\frac{1}{{\left(2\pi i\right)}^{2}}\int_{\upsilon_{1}}\int_{\upsilon_{2}}\Gamma \left(\upsilon_{1}\right)\frac{\Gamma \left(\upsilon_{2}\right)\Gamma \left(\upsilon_{2}+m\right)}{\Gamma (1+\upsilon_{2})}\\ \; & \times {\left(\mu_{\langle i\rangle }{\bar{\gamma }}_{I}\right)}^{\upsilon_{1}}{\left(\frac{{\bar{\gamma }}_{1}\left(\alpha_{c}-\left(\alpha_{p}+\kappa_{sr}^{2}\right)\gamma \right)}{m\gamma }\right)}^{\upsilon_{2}}\int_{0}^{\infty }\frac{x^{j-\upsilon_{1}-1}}{{\left(1+\left(1+\kappa_{Ir}^{2}x\right)\right)}^{\upsilon_{2}}}dxd\upsilon_{1}d\upsilon_{2} \end{array}$$

Finally, by employing ([25] Equations (3.194.3) and (8.384.1)) and performing some algebraic manipulations, we arrive at

(A6) $$\begin{array}{cc} F_{\gamma_{sr,c}}\left(\gamma \right) & =1-\sum_{i=1}^{\rho (R)}\sum_{j=1}^{\tau_{i}\left(R\right)}\frac{\chi_{i,j}\left(R\right)\vartheta_{1}^{-j}}{\Gamma (j)\Gamma (m)}\frac{1}{{\left(2\pi i\right)}^{2}}\int_{\upsilon_{1}}\int_{\upsilon_{2}}\Gamma \left(\upsilon_{1}+\upsilon_{2}-j\right)\Gamma \left(\upsilon_{1}\right)\\ \; & \times \Gamma \left(j-\upsilon_{1}\right)\frac{\Gamma (\upsilon_{2}+m)}{\Gamma (1+\upsilon_{2})}\vartheta_{1}^{\upsilon_{1}}\vartheta_{2}^{\upsilon_{2}}d\upsilon_{1}d\upsilon_{2} \end{array}$$

Therefore, by applying the definition of the BMGF [30], the final expression of $F_{\gamma_{sr,c}}\left(\gamma \right)$ is calculated as (19).

In addition, by transforming (7) to obtain $\gamma_{2}=\frac{\gamma }{\alpha_{c}-\left(\alpha_{p}+\kappa_{rd}^{2}\right)\gamma }$ and substituting it into the CDF expression of $\gamma_{2}$ in (15), the expression for $F_{\gamma_{rd,c}}\left(\gamma \right)$ is obtained as (20).

## Appendix B. Proof of the Expression of $F_{\gamma_{sr,c}}^{\infty }\left(\gamma \right)$

To derive the asymptotic CDF of $\gamma_{sr,c}$, we apply an appropriate identity transformation to rewrite the original BMGF in (A6) as a product of a Meijer’s G function and several auxiliary functions. By using ([33] Equation (07.34.02.0001.01)), (A6) can be reformulated as

(A7) $$F_{\gamma_{sr,c}}\left(\gamma \right)=1-\sum_{i=1}^{\rho (R)}\sum_{j=1}^{\tau_{i}\left(R\right)}\frac{\chi_{i,j}\left(R\right)\vartheta_{1}^{-j}}{\Gamma (j)\Gamma (m)}\frac{1}{2\pi i}\int_{\upsilon_{1}}\Gamma \left(\upsilon_{1}\right)\Gamma \left(j-\upsilon_{1}\right)G_{1,2}^{2,0}\left[\left.\begin{array}{c} \frac{1}{\vartheta_{2}} \end{array}\right|\begin{array}{c} 1\\ \upsilon_{1}-j,m \end{array}\right]\vartheta_{1}^{\upsilon_{1}}d\upsilon_{1}$$

When ${\bar{\gamma }}_{1}\to \infty$, the Meijer’s G function in (A7) is approximated by employing ([33] Equation (07.34.06.0040.01)) as

(A8) $$\begin{array}{c} G_{1,2}^{2,0}\left[\left.\begin{array}{c} \frac{1}{\vartheta_{2}} \end{array}\right|\begin{array}{c} 1\\ \upsilon_{1}-j,m \end{array}\right]\to \frac{\Gamma (m+j-\upsilon_{1})}{\Gamma (1+j-\upsilon_{1})}{\left(\frac{1}{\vartheta_{2}}\right)}^{\upsilon_{1}-j} \end{array}$$

Inserting (A8) into (A7) and applying ([33] Equations (07.34.02.0001.01) and (07.34.17.0011.01)), the expression of $F_{\gamma_{sr,c}}^{\infty }\left(\gamma \right)$ is formulated as

(A9) $$\begin{array}{c} F_{\gamma_{sr,c}}^{\infty }\left(\gamma \right)=1-\sum_{i=1}^{\rho (R)}\sum_{j=1}^{\tau_{i}\left(R\right)}\frac{\chi_{i,j}\left(R\right)}{\Gamma (j)\Gamma (m)}G_{2,2}^{2,1}\left[\left.\begin{array}{c} \frac{\vartheta_{1}}{\vartheta_{2}} \end{array}\right|\begin{array}{c} 1-j,1\\ 0,m \end{array}\right] \end{array}$$

From (A9), if ${\bar{\gamma }}_{I}$ is comparable to ${\bar{\gamma }}_{1}$, $F_{\gamma_{sr,c}}^{\infty }\left(\gamma \right)$ is derived as (27). Assuming a fixed ${\bar{\gamma }}_{I}$ and further letting ${\bar{\gamma }}_{1}\to \infty$ in (A9), $F_{\gamma_{sr,c}}^{\infty }\left(\gamma \right)$ is finally derived by applying ([33] Equation (07.34.06.0040.01)) as (28).

## Appendix C. Proof of the Expression for ${\bar{C}}_{sr,c}$

Due to the complexity of the BMGF, we suppose that *m* is an integer when computing the EC. By applying ([25] Equation (3.351.2)), $F_{\gamma_{1}}\left(\gamma \right)$ can be rewritten as

(A10) $$\begin{array}{cc} F_{\gamma_{1}}\left(\gamma \right) & =1-\sum_{s=0}^{m-1}\frac{1}{s!}{\left(\frac{m\gamma }{{\bar{\gamma }}_{1}}\right)}^{s}e^{-\frac{m\gamma }{{\bar{\gamma }}_{1}}} \end{array}$$

Thus, by inserting (A10) and (12) into (A2), $F_{\gamma_{sr,c}}\left(\gamma \right)$ is expressed as

(A11) $$\begin{array}{cc} F_{\gamma_{sr,c}}\left(\gamma \right) & =1-\sum_{s=0}^{m-1}\sum_{i=1}^{\rho (R)}\sum_{j=1}^{\tau_{i}\left(R\right)}\frac{\chi_{i,j}\left(R\right){\left(\mu_{\langle i\rangle }{\bar{\gamma }}_{I}\right)}^{-j}}{\Gamma (j)s!}{\left(\frac{1}{\vartheta_{2}}\right)}^{s}e^{-\frac{1}{\vartheta_{2}}}\\ \; & \times \int_{0}^{\infty }x^{j-1}{\left(1+\left(1+\kappa_{Ir}^{2}\right)x\right)}^{s}e^{-\left(\frac{\left(1+\kappa_{Ir}^{2}\right)}{\vartheta_{2}}+\frac{1}{\mu_{\langle i\rangle }{\bar{\gamma }}_{I}}\right)x}dx \end{array}$$

By applying the expansion 1+(1+κIr2)xs=∑t=0sstxt(1+κIr2)t ([25] Equation (1.111)), then using ([25] Equation (3.326.2)), and finally employing the identity $x^{s}e^{-x}=G_{0,1}^{1,0}\left[\left.\begin{array}{c} x \end{array}\right|\begin{array}{c} -\\ s \end{array}\right]$ ([33] Equation (07.34.03.0228.01)), (A11) can be transformed into

(A12) Fγsr,c(γ)=1−∑s=0m−1∑t=0s∑i=1ρ(R)∑j=1τi(R)stχi,j(R)ϑ1−jΓ(j)s!Γ(t+j)Φt+jG0,11,01ϑ2−s

where $\Phi =\frac{1}{\vartheta_{1}}+\frac{1}{\vartheta_{2}}$. When $\gamma >\frac{\alpha_{c}}{\alpha_{p}+\kappa_{sr}^{2}}$, ${\bar{C}}_{sr,c}=0$; while $\gamma <\frac{\alpha_{c}}{\alpha_{p}+\kappa_{sr}^{2}}$, ${\bar{C}}_{sr,c}$ is written as

(A13) $${\bar{C}}_{sr,c}=\frac{1}{\ln(2)}\int_{0}^{\frac{\alpha_{c}}{\alpha_{p}+\kappa_{sr}^{2}}}\frac{1-F_{sr,c}\left(\gamma \right)}{1+\gamma }d\gamma$$

By substituting (A12) into (A13) and performing the variable substitution $x=\frac{\gamma }{\alpha_{c}-\left(\alpha_{p}+\kappa_{sr}^{2}\right)\gamma }$, ${\bar{C}}_{sr,c}$ can be simplified as

(A14) C¯sr,c=1ln(2)∫0∞∑s=0m−1∑t=0s∑i=1ρ(R)∑j=1τi(R)stχi,j(R)ϑ1−jΓ(j)s!Γ(t+j)(1ϑ1+mxγ¯1)t+j×1+(αp+κsr2)x1+(1+κsr2)xG0,11,0mxγ¯1−sαc(1+(αp+κsr2)x)2dx

By utilizing the Gauss–Laguerre formula ([31] Equation (25.4.45)), the expression in (A14) can be approximated using numerical integration as shown in (36).
